# Supplementary material for: Modeling glioblastoma heterogeneity as a dynamic network of cell states
Source: Mol Syst Biol. 2021 Sep 16;17(9):e10105. doi: 10.15252/msb.202010105 (PMC8444284; doi:10.15252/msb.202010105)
Supplement: Supplementary file 6 — Source Data for Figure 5 [file MSB-17-e10105-s004.zip › Figure5A_sourcedata/GSEA_3017/hallmarks_stateF.GseaPreranked.1621934628066/HALLMARK_INTERFERON_GAMMA_RESPONSE.html]

Details for gene set HALLMARK\_INTERFERON\_GAMMA\_RESPONSE[GSEA]

|  || Dataset | state33017 |
| Phenotype | NoPhenotypeAvailable |
| Upregulated in class | na\_pos |
| GeneSet | HALLMARK\_INTERFERON\_GAMMA\_RESPONSE |
| Enrichment Score (ES) | 0.46509737 |
| Normalized Enrichment Score (NES) | 2.1829603 |
| Nominal p-value | 0.0 |
| FDR q-value | 0.0019777797 |
| FWER p-Value | 0.011 |
Table: GSEA Results Summary

  

Fig 1: Enrichment plot: HALLMARK\_INTERFERON\_GAMMA\_RESPONSE      
 Profile of the Running ES Score & Positions of GeneSet Members on the Rank Ordered List

  

| PROBE | GENE SYMBOL | GENE\_TITLE | RANK IN GENE LIST | RANK METRIC SCORE | RUNNING ES | CORE ENRICHMENT || 1 | CDKN1A |  |  | 4 | 2.089 | 0.1112 | Yes |
| 2 | CCL2 |  |  | 7 | 1.774 | 0.2064 | Yes |
| 3 | IFITM3 |  |  | 38 | 0.987 | 0.2462 | Yes |
| 4 | BST2 |  |  | 55 | 0.910 | 0.2883 | Yes |
| 5 | IFITM2 |  |  | 103 | 0.749 | 0.3076 | Yes |
| 6 | B2M |  |  | 110 | 0.736 | 0.3448 | Yes |
| 7 | NAMPT |  |  | 168 | 0.634 | 0.3534 | Yes |
| 8 | ST3GAL5 |  |  | 180 | 0.612 | 0.3815 | Yes |
| 9 | BTG1 |  |  | 222 | 0.574 | 0.3941 | Yes |
| 10 | PSMB8 |  |  | 230 | 0.568 | 0.4216 | Yes |
| 11 | C1R |  |  | 249 | 0.554 | 0.4435 | Yes |
| 12 | STAT2 |  |  | 267 | 0.540 | 0.4651 | Yes |
| 13 | VAMP5 |  |  | 341 | 0.491 | 0.4588 | No |
| 14 | LGALS3BP |  |  | 528 | 0.424 | 0.3978 | No |
| 15 | PARP14 |  |  | 541 | 0.417 | 0.4150 | No |
| 16 | IFIT2 |  |  | 624 | 0.391 | 0.3992 | No |
| 17 | IFIT1 |  |  | 720 | 0.365 | 0.3761 | No |
| 18 | FAS |  |  | 740 | 0.361 | 0.3871 | No |
| 19 | SOCS3 |  |  | 869 | 0.332 | 0.3473 | No |
| 20 | STAT3 |  |  | 880 | 0.330 | 0.3607 | No |
| 21 | ARL4A |  |  | 889 | 0.328 | 0.3748 | No |
| 22 | IRF9 |  |  | 892 | 0.327 | 0.3916 | No |
| 23 | HIF1A |  |  | 972 | 0.314 | 0.3730 | No |
| 24 | ARID5B |  |  | 1014 | 0.307 | 0.3711 | No |
| 25 | PELI1 |  |  | 1059 | 0.298 | 0.3674 | No |
| 26 | C1S |  |  | 1087 | 0.294 | 0.3712 | No |
| 27 | RNF213 |  |  | 1091 | 0.294 | 0.3857 | No |
| 28 | VCAM1 |  |  | 1266 | 0.272 | 0.3220 | No |
| 29 | TAP1 |  |  | 1317 | 0.266 | 0.3138 | No |
| 30 | PSMB9 |  |  | 1347 | 0.262 | 0.3149 | No |
| 31 | APOL6 |  |  | 1359 | 0.261 | 0.3241 | No |
| 32 | SP110 |  |  | 1384 | 0.258 | 0.3273 | No |
| 33 | PDE4B |  |  | 1404 | 0.256 | 0.3326 | No |
| 34 | UBE2L6 |  |  | 1415 | 0.255 | 0.3419 | No |
| 35 | METTL7B |  |  | 1704 | -0.320 | 0.2293 | No |
| 36 | UPP1 |  |  | 1726 | -0.328 | 0.2376 | No |
Table: GSEA details [plain text format]

  

Fig 2: HALLMARK\_INTERFERON\_GAMMA\_RESPONSE: Random ES distribution      
 Gene set null distribution of ES for **HALLMARK\_INTERFERON\_GAMMA\_RESPONSE**
